# Efficacy and Safety of Nerinetide in Acute Ischemic Stroke Patients: A Systematic Review, Meta‐Analysis and Meta Regression

**DOI:** 10.1002/brb3.71049

**Published:** 2025-11-10

**Authors:** Laiba Khurram, Laksh Kumar, Muaz Noor, Faizan Shams, Muhammad Yousuf, Abdullah Ubaid, Jodho Ji, FNU Somdev, FNU Samiullah, Muhammad Junaid Imran, Waqar Malik, Adarsh Raja, Abdullah BS, Biruk Demisse Ayalew, Aayush Chaulagain

**Affiliations:** ^1^ Department of Internal Medicine Shaheed Mohtarma Benazir Bhutto Medical College Lyari Karachi Pakistan; ^2^ Department of Internal Medicine Karachi Metropolitan University Karachi Pakistan; ^3^ Department of Medicine Rawalpindi Medical University Rawalpindi Punjab Pakistan; ^4^ St. Paul's Hospital Millennium Medical College Addis Ababa Ethiopia; ^5^ Patan Academy of Health Sciences Lalitpur Nepal

**Keywords:** acute ischemic stroke, functional recovery, meta‐analysis, mortality, nerinetide, neuroprotection

## Abstract

**Purpose:**

Acute ischemic stroke (AIS) is a leading cause of disability and death worldwide. Nerinetide, a neuroprotective peptide, may limit ischemic brain injury. This systematic review and meta‐analysis evaluated the efficacy and safety of nerinetide versus placebo in patients with AIS.

**Method:**

Following the PRISMA and AMSTAR 2 guidelines, PubMed, MEDLINE, Cochrane Library, ScienceDirect, and ClinicalTrials.gov were searched until July 2025 for randomized controlled trials (RCTs) involving adult patients with AIS. The primary outcome was mortality, and secondary outcomes included functional recovery (modified Rankin Scale [mRS] scores of 0–1 and 0–2 and Barthel index scores of ≥95), infarct volume, and adverse events. Pooled risk ratios (RR) or mean differences (MD) were calculated using a random‐effects model, and meta‐regression was used to explore baseline moderators.

**Findings:**

Three RCTs (*n* = 2462) were included in the analysis. Nerinetide showed a borderline significant mortality reduction versus placebo (RR = 0.86; 95% CI 0.75–1.00; *p* = 0.05) with low heterogeneity (*I* ^2^ = 11%). No significant differences were found in functional recovery or overall infarct volume, although subgroup analyses without reperfusion therapy suggested a possible benefit. The rates of adverse events, including serious events, thromboembolism, seizures, and hypotension, were similar between the groups. Meta‐regression indicated that atrial fibrillation (*p* = 0.0489) and prior stroke (*p* = 0.0519) were associated with a higher mortality risk.

**Conclusion:**

Nerinetide may modestly reduce mortality in AIS without increasing adverse events but does not significantly improve the functional outcomes. The benefits may be greater in patients who do not receive thrombolysis. Further large‐scale trials are warranted to clarify the optimal use and interactions with reperfusion therapies.

## Graphical abstract

Nerinetide modestly reduced mortality in acute ischemic stroke (RR = 0.86; *p* = 0.05) without improving functional recovery (mRS, Barthel Index, infarct volume). Safety was comparable to placebo. Further large RCTs are needed to clarify its role and interaction with reperfusion therapies.

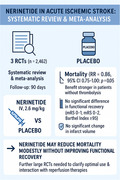

## 1 Introduction

Acute ischemic stroke (AIS) is a serious illness that arises when blood flow to certain sections of the brain is diminished or ceases, hindering oxygen and nutrition supply to the brain tissue, resulting in cell injury or death. Ischemic stroke is the second major cause of death and the third greatest cause of combined lifelong disability and fatality worldwide (Wang et al. 2024). Stroke costs over US$890 billion globally (0.66% of the global GDP) (Feigin et al. 2025). Despite having effective approaches to the treatment of AIS, about 50% of the patients experience disability after stroke (Houkin et al. 2024). Stroke‐related disabilities occur more frequently in low‐ and middle‐income countries than in high‐income countries (Feigin et al. 2023). This indicates that there is still room for intervention to achieve more productive outcomes.

Although AIS is a life‐threatening medical condition, it can be prevented and cured. Change in lifestyle helps in preventing stroke because there exist numerous alterable contributors, including hypertension, smoking, sedentary lifestyle etc. (Feigin et al. 2025). Conventional AIS therapies aim to restore blood flow to affected tissues. Reperfusion therapies include intravascular thrombolysis (IVT) a pharmacological procedure in which clot‐dissolving agents like alteplase, tenecteplase are administered into blood vessels to dissolve the clot, and endovascular thrombectomy (EVT) a mechanical technique used to retrieve clot, are the mainstay of treatment options for patients with AIS (Dammavalam et al. 2024). Nerinetide is an eicosapeptide administered to achieve neuroprotection for AIS (Mayor‐Nunez et al. 2021). It mitigates neurotoxicity triggered by neural nitric oxide synthase (nNOS) in AIS by minimizing the interaction between *N*‐methyl‐D‐aspartate receptors (NMDARs) and post‐synaptic density protein (95) in AIS (Zhou 2021). When nerinetide alone administered in AIS patients due to large vessel occlusion undergoing EVT there was improved functional outcome (Baron 2021).

This systematic review and meta‐analysis aimed to compare the safety and efficacy of nerinetide with that of placebo in individuals with AIS. This innovative meta‐analysis will address the gap in the literature, resulting in advancements in neurology and medicine.

## 2 Method

The present systematic review and meta‐analysis were performed in alignment with the AMSTAR 2 tool and the PRISMA guidelines, which offer structured recommendations for conducting and reporting systematic reviews and meta‐analyses (Page et al. 2021). These standards are intended to guarantee clarity and comprehensiveness in the reporting of systematic reviews to ensure the dependability and reproducibility of study outcomes. Our study's findings are more reliable because we followed the PRISMA standards, which guarantee a thorough, transparent, and methodologically sound review.

### 2.1 Data Bases and Search Strategy

A comprehensive electronic search was conducted using five databases, PubMed, Cochrane Library, MEDLINE, ScienceDirect, and ClinicalTrials.gov, from their earliest record to July 2025. The search aimed to identify studies involving human participants that compared the outcomes of nerinetide and AIS. No language restrictions or filters were applied to the search. A comprehensive search strategy is available in Table S1.

### 2.2 Study Selection and Eligibility Criteria

The studies extracted from the database search were deduplicated using Mendeley software. Subsequently, a thorough screening process was conducted, beginning with a title and abstract search and progressing to a rigorous full‐text examination of each applicable paper. Studies ought to be screened autonomously by at least two distinct individuals (or person/machine combination) with a procedure to settle disagreements. The exclusion criteria were as follows: (1) Non RCTS, (2) studies including pediatric patients, and (3) studies not reporting outcomes of interest. Studies conducted in regions where nerinetide is not approved or available will also be excluded unless they meet all other inclusion criteria. The inclusion criteria were as follows: (1) studies reporting patients with AIS; (2) original studies; (3) studies including a direct comparison of nerinetide versus placebo; and (4) studies reporting at least one outcome of interest.

### 2.3 Data Extraction and Outcomes

Key variables will be compiled autonomously by at least two individuals to maintain precision and minimize bias. Discrepancies between data extractors will be addressed through discussion, and if unresolved, by involving a third reviewer. In cases where critical data are unavailable in the published articles, the original study authors will be contacted to obtain missing information, where possible, individual participant data (IPD) or full study datasets will be requested from the investigators or accessed via reputable data repositories. The primary outcomes of interest included death. Secondary outcomes included excellent and good recovery, defined as mRS scores of 0–1 and 0–2, respectively, a Barthel Index score of ≥95 and worsening of stroke. The safety outcomes to be assessed include the occurrence of any serious adverse events, stroke in evolution, hypotension, recurrent ischemic stroke, seizures, and pulmonary embolism or deep vein thrombosis.

### 2.4 Risk of Bias and Quality Assessments

The risk of bias in the included studies will be weighed using the Cochrane Risk of Bias 2.0 (RoB 2) method, which explores key domains such as randomization, blinding, shifts from the planned interventions, insufficient outcome data, and selective reporting (Sterne et al. 2019). A minimum of two reviewers or one reviewer supported by a machine‐assisted approach will independently evaluate each study article. Disagreements will be settled through discussion or, if needed, by involving a third reviewer. The primary investigators of the original studies will be approached if additional details are required. The risk of reporting bias due to insufficient results will also be evaluated. The overall reliability of the evidence for each outcome will be assessed.

### 2.5 Statistical Analysis

Statistical analyses were performed using RevMan software (version 5.4.1; The Nordic Cochrane Center, The Cochrane Collaboration, Copenhagen, 2020). The Mantel–Haenszel method was employed to determine risk ratios (RR) for dichotomous outcomes, whereas mean differences (MD) for continuous outcomes were estimated using the generic inverse variance method. Additionally, studies were stratified according to use of other concomitant treatment that is, use of alteplase and studies without reperfusion. Both results were reported with their respective 95% confidence intervals (CIs). A random‐effects model was employed to pool outcomes, and heterogeneity was assessed using Higgins “*I*” statistic. The degree of heterogeneity was interpreted as low when *I* ^2^ was below 50%, moderate between 50% and 75%, and high when exceeding 75% (Higgins et al. 2003). For outcomes exhibiting heterogeneity, meta‐regression was performed using comprehensive meta‐analyses (CMA) (Version 3.3.070) on baseline moderators, including mean age, atrial fibrillation, diabetes, peripheral vascular disease, any past stroke, hypertension, and hyperlipidemia. Statistical significance was set at *p* ≤ 0.05.

## 3 Results

### 3.1 Study Selection

**FIGURE 1:**
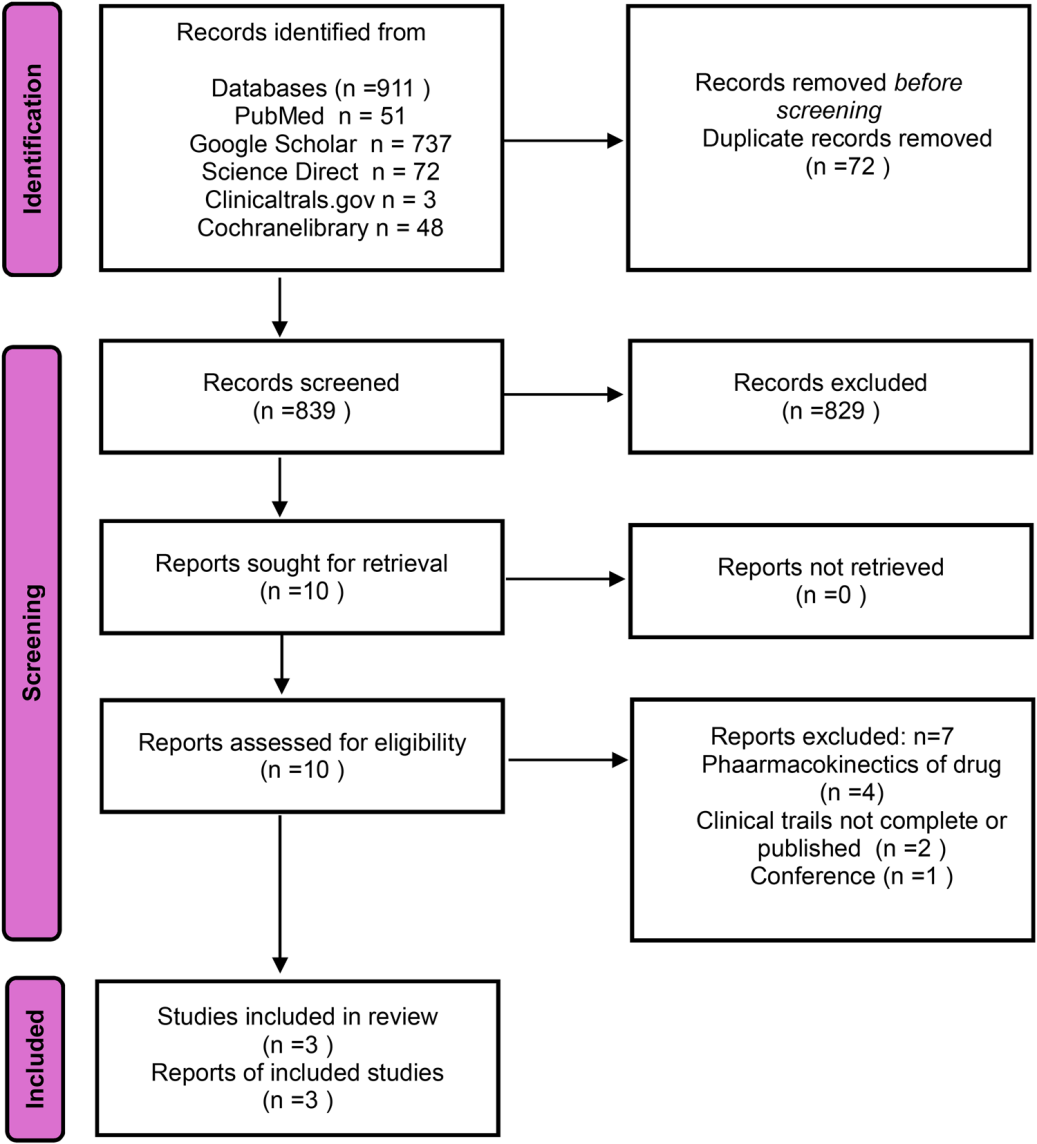
Prisma flow chart.

An initial comprehensive search of multiple databases and registries identified 911 records. After removing 72 duplicates, 839 records remained for title and abstract screening. Of these, 829 were excluded for not meeting the inclusion criteria. The full texts of the remaining 10 articles were retrieved and assessed for their eligibility. Seven articles were excluded: four pharmacokinetic studies, two incomplete or unpublished clinical trials, and one conference abstract. Ultimately, three studies fulfilled all eligibility criteria and were included in the final meta‐analysis (Figure 1).

### 3.2 Baseline Characteristics of Included Studies

Three randomized controlled trials comprising 2462 patients were included in this meta‐analysis. All patients received nerinetide at a dosage of 2.6 mg/kg. The ESCAPE‐NA1 trial by Michael et al. (2020) enrolled 1105 patients (549 received nerinetide and 556 received placebo), with a mean age of approximately 71 years (Hill et al. 2020). Christenson et al. (2025) conducted a trial involving 507 patients (254 in the nerinetide group, 253 in placebo), with an average age of 74–75 years. The most recent trial by Hill et al. (2025) included 850 patients, with 454 receiving nerinetide and 396 placebo, and a mean age of 75–76 years (Christenson et al. 2025). The male‐to‐female ratio was generally balanced across the studies. The follow‐up duration in all three trials was 90 days. Baseline comorbidities such as hypertension, diabetes, and atrial fibrillation were present in both groups across all studies in relatively similar proportions Table 1.

**TABLE 1:** Study and baseline patient characteristics.

				Patients			Age (years)		Sex		Any past stroke		Congestive heart failure		Peripheral vascular disease		Hyperlipidaemia		Atrial fibrillation		Diabetes		Renal disease		Hypertension		
Study name	Clinical trial no.	Year of study	Total sample	**Nerinetide**	**Placebo**	Dosage Nerinetide	**Nerinetide**	**Placebo**	**Nerinetide (M/W)**	**Placebo (M/W)**	**Nerinetide**	**Placebo**	**Nerinetide**	**Placebo**	**Nerinetide**	**Placebo**	**Nerinetide**	**Placebo**	**Nerinetide**	**Placebo**	**Nerinetide**	**Placebo**	**Nerinetide**	**Placebo**	**Nerinetide**	**Placebo**	Follow up time period
Michael et al. (2020)	NCT02930018.	2020	1105	549	556	2.6 mg/kg,	71.5 ± 13.8	70.3 ± 14.6	281/268	275/281	81 (14.8%)	76 (13.7%)	72 (13.1%)	65 (11.7%)	31 (5.7%)	28 (5.1%)	254 (46.3%)	260 (46.9%)	195 (35.5%)	192 (34.6%)	111 (20.2%)	107 (19.3%)	35 (6.4%)	28 (5.1%)	378 (68.9%)	396 (71.4%)	90 days
Christenson et al. (2025)	NCT02315443	2025	507	254	253	2.6 mg/kg,	74 ± 11.85	75 ± 14.07	147/107	141/112	23 (9%)	18 (7%)	22 (9%)	28 (11%)	16 (6%)	29 (11%)	124 (49%)	123 (49%)	73 (29%)	64 (25%)	63 (25%)	56 (22%)	37 (15%)	34 (13%)	186 (73%)	181 (72%)	90 days
Hill et al. (2025)	NCT04462536.	2025	850	454	396	2.6 mg/kg,	76 ± 12.59	75 ± 13.33	228/226	201/195	95/451 (21%)	84/396 (21%)	75/451 (17%)	57/396 (14%)	32/451 (7%)	30/396 (8%)	219/451 (49%)	192/395 (49%)	228/451 (51%)	207/396 (52%)	107/451 (24%)	89/396 (22%)	68/451 (15%)	45/396 (11%)	362/451 (80%)	299/396 (76%)	90 days

### 3.3 Primary Outcome

#### 3.3.1 Deaths

**FIGURE 2:**
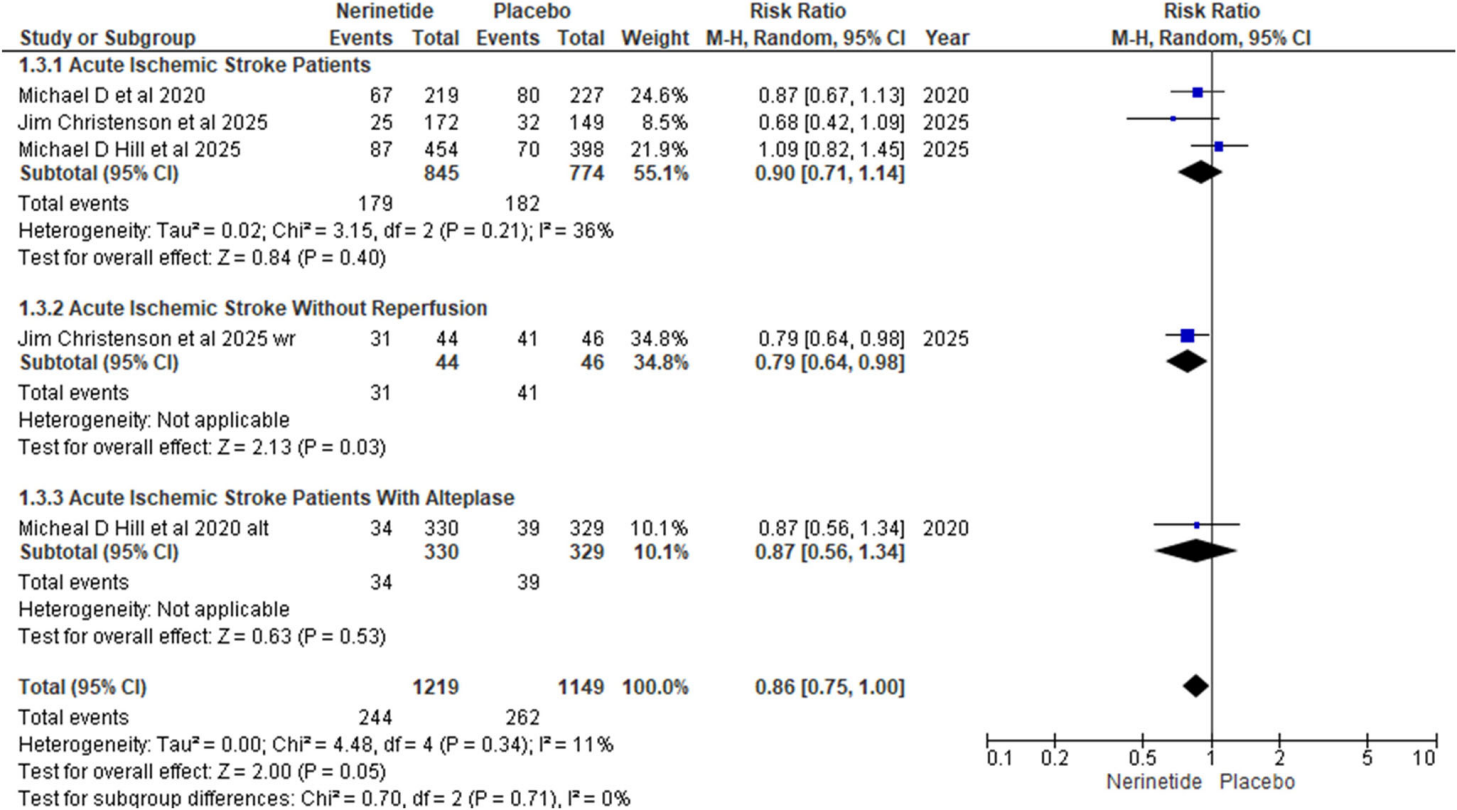
Forest plot for deaths.

We formed subgroups to evaluate the risk of death in the nerinetide arm compared to that in the placebo arm. In the subgroup of AIS patients three studies Michael et al. (2020), Christenson et al. (2025) and Hill et al. (2025) show no significant difference in death outcome in nerinetide consumers in comparison to placebo (R.R: 0.9; 95% CI [0.71, 1.14]; *p*‐value:0.4). In contrast, in a study by Christenson et al. (2025) on patients with AIS without reperfusion, a marked reduction in death risk was observed in nerinetide consumers versus placebo (R.R: 0.79; 95%CI [0.64, 0.98]; *p* = 0.03). In patients with AIS treated with alteplase, one RCT demonstrated a similar risk of death in both groups (R.R: 0.87; CI [0.56,1.34]; *p* = 0.53). Overall pooled analysis showed a conclusive decline in the risk of death in the nerinetide population compared with placebo (R.R: 0.86; 95%CI [0.75,1.00]; *p* = 0.05) with non‐significant and low heterogeneity (*I* ^2^ = 11%; *p* = 0.34) (Figure 2).

### 3.4 Secondary Outcomes

#### 3.4.1 mRS 0–1

**FIGURE 3:**
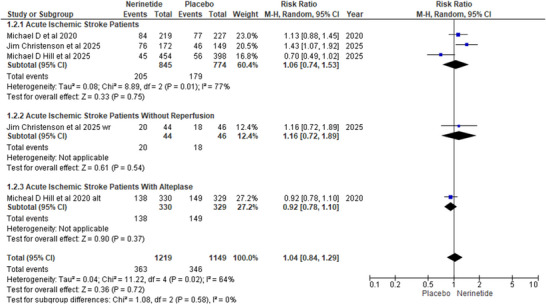
Forest plot for mRS 0–1.

The modified ranking scale of 0–1, excellent functional recovery in nerinetide recipients versus placebo recipients, was assessed by forming three subgroups. In the first cohort of AIS patients, three RCTS demonstrated no clinically significant benefit in the nerinetide arm compared to placebo (R.R: 1.06; 95% CI [0.74,1.53]; *p*‐value: 0.75). A similar result was found in the subgroup without reperfusion (R.R, 1.01; 95% CI [0.72, 1.89]; *p* = 0.54). However, AIS patients treated with alteplase showed a non‐significant negative trend in the nerinetide‐stratified arm (R.R, 0.92; 95% CI [0.78, 1.10]; *p* = 0.37). The overall effect in all three stratified subgroups was not found to reach statistically significant (R.R, 1.04; 95%CI [0.84, 1.29]; *p* = 0.58) with marked heterogeneity (*I* ^2^ = 64%; *p* = 0.02) (Figure 3).

#### 3.4.2 mRS 0–2

**FIGURE 4:**
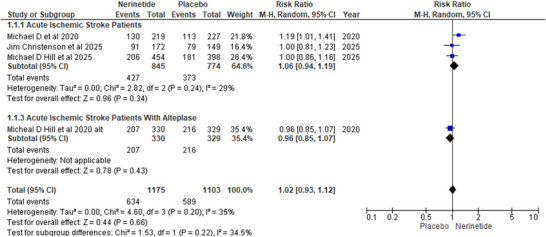
Forest plot for mRS 0–2.

The modified ranking scale of 0–2, good recovery in the nerinetide arm stratified population in relation to placebo was analyzed, forming two subgroups. In the first subgroup of AIS patients, three studies reported insignificant benefits in the nerinetide population versus placebo (R.R: 1.06; 95%CI [0.95, 1.19]; *p* = 0.34). Similarly, a statistically non‐significant trend was demonstrated by the second cohort of AIS patients taking alteplase (R.R, 0.96; CI [0.85,1.07]; *p* = 0.43). Overall pooled analysis showed no difference in mRS 0–2 outcome in both arms (R.R: 1.02; 95%CI [0.93,1.12]; *p* = 0.66) with an insignificant value of heterogeneity (*I* ^2^ = 35%; *p* = 0.2) (Figure 4).

#### 3.4.3 Infarct Volume

We estimated the size of the infarct using the infarct volume parameter in nerinetide recipients compared to placebo recipients. We defined two subgroups for analysis, in the subgroup of AIS patients, three RCTs, including Michael et al. (2020); Christenson et al. (2025); Hill et al. (2025), showed a notable reduction in infarct size in nerinetide‐stratified patients in contrast to placebo stratified patients(MD = −10.70; CI [−17.87,−3.53]; *p* = 0.003).In contrast, in the cohort of AIS patients treated with alteplase, there was no difference in infarct volume between the nerinetide and placebo groups. In conclusion, the overall effect size indicated no meaningful variation in infarct size between the nerinetide and placebo arms (MD = −6.7;95%CI [−14.9,0.77]; *p* = 0.08) with mild heterogeneity (*I* ^2^ = 42%; *p* = 0.16) (Figure S2A).

#### 3.4.4 Barthel Index (≥95)

Barthel index ≥95 was used to evaluate near‐complete functional independence, and three subgroups were formed to conduct an analysis. The first subgroup, AIS patients, including three RCTs suggested comparable amounts of functional independence in both nerinetide recipients versus placebo recipients (R.R: 1.02; 95% CI [0.91,1.13]; *p* = 0.90), and AIS patients without reperfusion revealed slight but insignificant improvement in functional independence in nerinetide recipients compared to placebo recipients (R.R: 1.16; 95%CI [0.72, 1.89]; *p* = 0.54). AIS patients taking alteplase showed no statistically significant improvement in Barthel index score in the nerinetide population (R.R: 1.16; 95%CI [0.93, 1.15]; *p* = 0.55). The overall effect of all the subgroups illustrated that nerinetide failed to impact on Barthel index score (R.R: 1.02; 95%CI [0.95, 1.10]; *p* = 0.55) with no amount of heterogeneity (*I* ^2^ = 0%; *p* = 0.67) (Figure S2B).

#### 3.4.5 Worsening of Stroke

To analyze the worsening of stroke outcomes, we stratified the studies into two subgroups: the AIS patients subgroup, containing three RCTs, suggested non‐significant findings (R.R: 0.75; 95%CI [0.39,1.41], *p* = 0.37).In the second subgroup, with no reperfusion history, a study by Christenson et al. (2025) illustrated an equivalent amount of risk in both nerinetide and placebo. The final analysis demonstrated an insignificant decreased risk of worsening of stroke (R.R: 0.83; 95% CI [0.56,1.22]; *p* = 0.34) with no significant heterogeneity (*I* ^2^ = 45%; *p* = 0.16) (Figure S2C).

#### 3.4.6 Stroke in Evolution

All three RCTs reported this outcome. The overall pooled effect demonstrated an insignificant decline in the risk of stroke progression in the nerinetide population compared to placebo (R. R:0.9; 95%CI [0.66,1.22]; *p* = 0.5). No heterogeneity was recorded (*I* ^2^ = 0%; *p* = 0.85) (Figure S2D).

### 3.5 Recurrent or New Ischemic Stroke

Three RCTs reported this outcome. The overall pooled effect showed an insignificant increase in the risk of new str% *p* episodes in the nerinetide stratified population in comparison with placebo, with moderate but insignificant heterogeneity (R.R: 1.41; 95%CI [0.68,2.94]; *p* = 0.35); (*I* ^2^ = 50%; *p* = 0.14) (Figure S2E).

#### 3.5.1 Hypotension

This outcome was reported by two RCTs, Michael et al. (2020) and Christenson et al. (2025), and the overall analysis suggested a negligible difference in the risk of hypotension in nerinetide and placebo recipients (R.R: 1.66; 95%CI [0.12,23.53]; *p* = 0.71). There was a significant amount of heterogeneity (*I* ^2^ = 78%; *p* = 0.03) (Figure S2F).

### 3.6 Any Serious Adverse Events

All three RCTs reported this outcome, and no meaningful difference was observed in nerinetide‐taking patients in relation to placebo (R.R: 1.17; 95%CI [0.85,1.6]) high amount of heterogeneity was found among these three studies (*I* ^2^ = 89%; *p* ≤ 0.0001) (Figure S2G).

#### 3.6.1 Seizures

Hill et al. (2025) and Christenson et al. (2025) reported this outcome. The overall pooled effect showed a slight, although insignificant rise in events of seizures in the nerinetide population in relation to placebo with no heterogeneity (R.R: 1.56; 95%CI [0.76,0.32]) (*I* ^2^ = 0%; *p* = 0.52) (Figure S2H).

### 3.7 Pulmonary Embolism or Deep Vein Thrombosis

All three RCTs reported these events. The overall pooled effect was comparable in the nerinetide population versus placebo, suggesting an equal risk of thromboembolic events in both groups (R.R: 1.16; 95%CI [0.25,5,46] *p* = 0.85). Insignificant but moderate heterogeneity was indicated by the analysis (*I* ^2^ = 78%; *p* = 0.07) (Figure S2I).

#### 3.7.1 Meta Regression

Meta regression was performed to assess the impact of baselines moderators that is, hyperlipidaemias, HTN, atrial fibrillation, any past stroke, diabetes, peripheral vascular disease, and mean age, on our primary outcome, death. Among them, atrial fibrillation showed a positive association with the risk of death (coefficient = 0.0142; *p* = 0.0489), while any past stroke also demonstrated a positive and marginally significant association (coefficient = 0.0259; *p* = 0.0519). All other moderators illustrated insignificant impact on the death outcome hyperlipidaemias (coefficient = −0.0033; *p* = 0.95440), HTN (coefficient = 0.0329; *p* = 0.1611), diabetes (coefficient: −0.0051; *p* = 0.1423), peripheral vascular disease (coefficient = −0.0297; *p* = 0.546), mean age (coefficient = 0.0121; *p* = 0.7338) (Figure S1A–G).

### 3.8 Risk of Bias Assessment

The risk of bias in the three RCTs was determined using the Cochrane Risk of Bias tool. Across most categories, including blinding methods, allocation concealment, randomization, and outcome reporting, each trial demonstrated methodological rigor with a consistently low risk of bias. However, Christenson et al. (2025) showed an unknown risk of attrition bias. The overall integrity of the trial was not significantly compromised, but this raises the possibility of bias; hence, the results of this study should be interpreted with caution. The pooled meta‐analytic results were more credible and reliable because the other two trials, Michael et al. (2020) and Hill et al. (2025), showed minimal risk of bias (Figure 5A, 5B and Table S2).

**FIGURE 5A:**
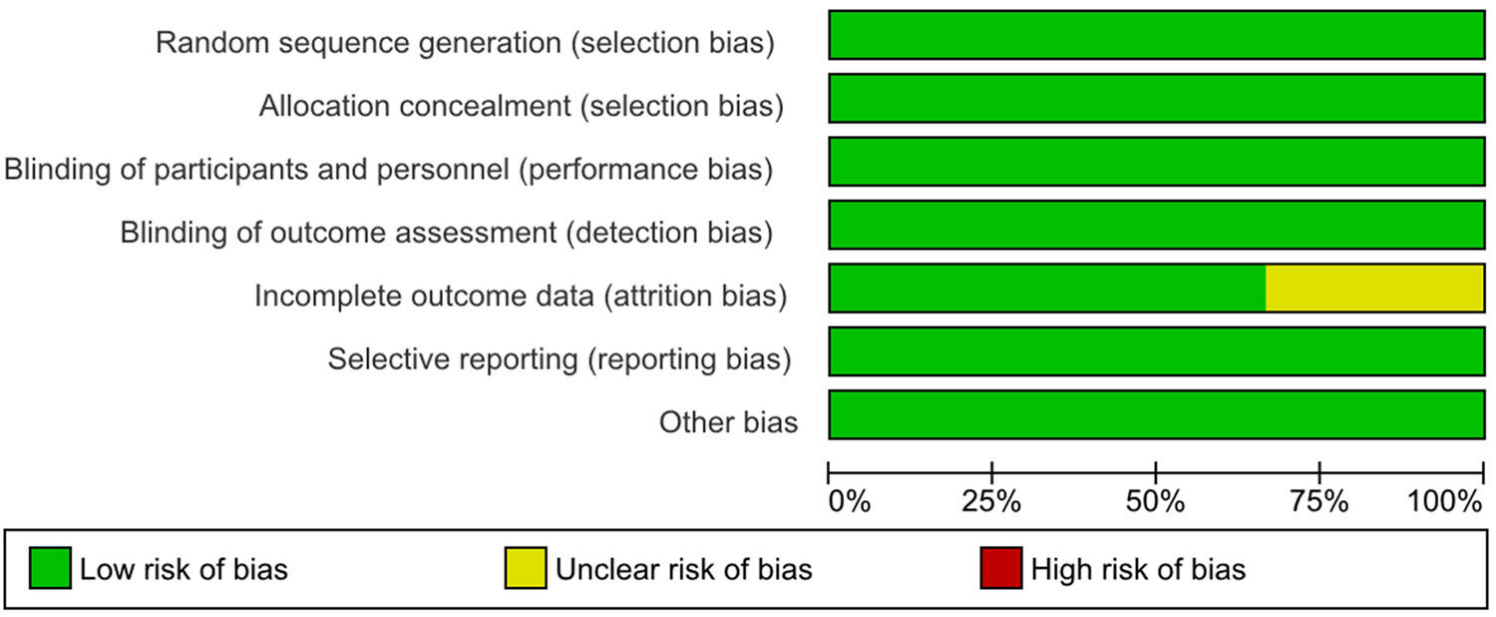
Risk of bias graph.

**FIGURE 5B:**
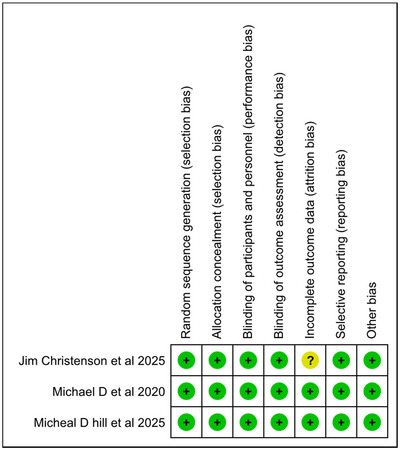
Risk of bias summary.

## 4 Discussion

Our meta‐analysis included three RCTs comprising a total of 2462 patients to evaluate the safety and efficacy of intravenous nerinetide compared with placebo in patients with AIS. The primary outcome mortality, defined as a modified Rankin Scale (mRS) score of 6, reached only borderline clinical significance with the pooled analysis indicated an approximate 14% relative reduction in deaths in the nerinetide group compared with the control group for key secondary functional outcomes, including functional independence measured by mRS 0–2 and mRS 0–1, as well as Barthel Index scores ≥95, no statistically significant differences were found between both arms. Notably, nerinetide treatment was not associated with a reduction in the risk of worsening of stroke and also demonstrated no significant change in infarct volume, suggesting a limited neuroprotective effect at the tissue level, although these signals did not consistently translate into improved functional independence. While these findings may indicate no clinical superiority of nerinetide over placebo, they should be critically analyzed. The lack of statistical significance does not necessarily imply no clinical benefit; instead, it underscores the need for further research into patient enrollment strategy, procedural elements, and outcome assessment in this population.

Current therapies for AIS focus on revascularization, either through fibrinolysis with alteplase or tenecteplase (both recombinant tissue plasminogen activators) or by EVT. Despite these approved therapies, a majority of patients remain functionally disabled after experiencing an AIS (Nimjee and Hill 2025). Defining the optimal neuroprotection drug strategy involves considering the drug target, appropriate patient population, optimal dose, temporal administration from stroke onset, and potential drug–drug interactions with standard‐of‐care AIS treatment. According to the current guideline, there are no FDA‐approved neuroprotectants for AIS treatment. In Asia, some putative neuroprotective agents are available and prescribed for stroke, including edaravone, dexborneol, and butyl phthalide (Li et al. 2024). Edaravone, an antioxidant and dexborbeol, a stereoisomer of borneol having both antioxidant and anti‐inflammatory properties, both demonstrated an improvement in functional outcome in different trials (Sharma et al. 2011; Fu et al. 2024). Similarly, butylphthalide, a substance derived from celery seeds, also showed promising results in reducing functional dependence at 90 days, particularly in AIS‐affected patients who concomitantly received thrombolytic therapy (Wang et al. 2023). However, the existing randomized trial evidence for the efficacy of these agents is unconvincing.

The majority of patients enrolled in our study had atrial fibrillation. Functional outcomes, assessed by the proportion of patients achieving an mRS score of 0–2, showed non‐significant numerical trends toward nerinetide. Therefore, there is no clear evidence that nerinetide improved functional independence at 90 days. This underscores the sustained challenges in patients regaining functional independence at 90 days, in contrast to the primary goal of acute stroke therapy to maximize recovery and minimize long‐term disability. Primarily, achieved by restoring blood flow and protecting the penumbra (an area of potentially reversible brain tissue injury caused by hypoperfusion due to stroke) from excitotoxicity that occurs due to overactivation of NMDARs (Ugalde‐Triviño and Díaz‐Guerra 2021).

Nerinetide, a peptide extension of the Discs large family of proteins (Zhu et al. 2016), perturbs the protein–protein interaction of postsynaptic density protein 95 (PSD‐95) with NMDA receptors and neuronal nitric oxide synthase. This minimizes excitotoxic signaling that could potentially result in ischemic neuronal death (Fraser et al. 2024). As an eicosapeptide, nerinetide disrupts the PSD‐95 signaling pathway, diminishing excitotoxic cell death in acute ischemic stroke.

A similar trend was observed for the more stringent endpoint of mRS 0–1, reflecting near‐complete recovery, although the effect size appeared smaller and may not have reached statistical significance in all comparisons. The Barthel index ≥95 indicated no statistically significant difference, although the numbers did imply a probable benefit. These clinical measures were further substantiated by a reduction in infarct volume on imaging, providing biological plausibility for the neuroprotective mechanism of nerinetide through its modulation of excitotoxic pathways.

An interesting finding from original research (Rex et al. 2024) is that nerinetide only reduces infarct growth in patients not treated with alteplase. This finding reinforces the hypothesis that nerinetide may fail to retain its neuroprotective efficacy due to protease‐mediated degradation, specifically by plasmin, a breakdown product of alteplase (Mayor‐Nunez et al. 2021). Nevertheless, there is optimism that a modified, plasmin‐resistant formulation of nerinetide may overcome this limitation in future applications (Mayor‐Nunez et al. 2021).

The non‐significant outcomes observed in certain nerinetide comparisons can be attributed to multiple factors, both pharmacological and methodological. A key limitation was the interaction between nerinetide and alteplase, as discussed above. Timing of administration also played a critical role; shortened intervals between infusion and reperfusion may have limited nerinetide's therapeutic reach, particularly in rapid workflows (Stoll et al. 2024). Patient heterogeneity, such as variability in infarct size, collateral circulation, and baseline comorbidities, could have masked treatment effects, especially in those with low ASPECTS or poor collateral scores (Mayor‐Nunez et al. 2021). Moreover, stringent outcome measures like mRS 0–1 may not fully capture functional gains. This discrepancy between literature and clinical results is illustrated by a number of confounding factors. The aforementioned combination with alteplase, which minimizes nerinetide's efficacy, is one of the major concerns (Rex et al. 2024). Furthermore, the therapeutic window may be shortened due to limited infusion‐to‐reperfusion periods in current EVT workflows, which might not allow for standard drug exposure prior to reperfusion injury (Hill et al. 2020). In particular groups, baseline moderators such as comorbidities, infarct size, and atrial fibrillation may introduce hindrance in achieving desired clinical results (Patel and Bhaskar 2023). Finally, relying on dichotomized endpoints such as mRS 0–1 or 0–2 might underestimate more subtle but clinically significant shifts in recovery that ordinal or utility‐weighted analyses could otherwise explain better (Tokunboh et al. 2022). After taking into account all these factors, we get a possible explanation of why the functional outcome failed to attain statistically significant improvement despite a credible pathophysiological rationale. Furthermore, future trials should include the data of patients receiving nerinetide as a sole drug to avoid possible drug interactions. Additionally, patients should be stratified in trials according to their comorbidities, such as atrial fibrillation and history of previous stroke to overcome the concern of heterogeneity allowing to study the details of their influence on the clinical outcome of nerinetide arm (Hart and Pearce 2009). Development of plasmin resistant nerinetide formulation will minimize the concern of plasmin mediated degradation of the drug (Mayor‐Nunez et al. 2021). Moreover, there is a need for large multi‐center trials for better generalizability of the findings (Rothwell 2005). Last, as stated in the Frontier trial, immediate administration of the drug necessitates additional evaluation before employing reperfusion strategies (Christenson et al. 2025).

The safety profile of nerinetide continues to appear acceptable based on prior randomized controlled trials. Studies, such as ESCAPE‐NA1 (Hill et al. 2020) and ESCAPE‐NEXT (Hill et al. 2025) reported no significant difference in serious adverse events. However, our meta‐analysis revealed a numerically greater incidence of serious adverse events among patients treated with nerinetide. Although this difference did not reach statistical significance, the trend warrants attention. It may represent early signals that were underpowered in individual studies but more perceptible when pooled across larger populations. Importantly, specific adverse outcomes such as pulmonary embolism, deep vein thrombosis, or hypotension did not show a statistically meaningful increase, consistent with the overall safety profile.

### 4.1 Limitation

This meta‐analysis has several limitations that should be acknowledged. First, the number of available randomized controlled trials was limited, and some individual studies had relatively small sample sizes. Second, there was notable heterogeneity in some key pooled outcomes, including mRS 0–1, any serious adverse events, hypotension, and infarct volume. This clinical and statistical heterogeneity likely reflects variations in patient selection, timing of nerinetide administration, concomitant treatments such as alteplase, and differences in study protocols. Third, this meta‐analysis relied on aggregated published data rather than individual patient‐level data, which restricts the ability to adjust for confounders or conduct detailed subgroup analyses. Finally, the potential for publication bias cannot be ruled out, as studies with negative or inconclusive results may remain unpublished.

## 5 Conclusion

This meta‐analysis suggests that intravenous nerinetide may offer a statistically significant reduction in mortality in patients with acute ischemic stroke compared to placebo. However, most secondary outcomes, including infarct volume reduction and functional improvement, did not reach statistical significance. The safety profile appears generally acceptable, though a slight numerical increase in serious adverse events was observed. These findings suggest a potential survival benefit but raise questions regarding the clinical meaningfulness of nerinetide in terms of functional recovery. Further large‐scale trials are warranted to clarify its therapeutic role, optimal timing, and interaction with thrombolytics. Until more definitive evidence is available, routine clinical use of nerinetide remains premature.

## Author Contributions

**Laiba Khurram**: conceptualization, project development, data collection, manuscript writing. **Laksh Kumar**: project development, data collection, manuscript writing. **Muaz Noor**: project development, data collection, manuscript writing. **Faizan Shams**: project development, data collection, manuscript writing. **Muhammad Yousuf**: table, manuscript writing. **Abdullah Ubaid**: data analysis, manuscript writing. **Jodho Ji**: data analysis, manuscript writing. **FNU Somdev**: figures, data analysis, manuscript writing. **FNU Samiullah**: figures, manuscript writing. **Muhammad Junaid Imran**: manuscript writing and editing. **Waqar Malik**: supervision, manuscript writing, and editing. **Adarsh Raja**: manuscript writing and editing. **Abdullah BS**: supervision, manuscript writing, and editing. **Biruk Demisse Ayalew**: manuscript writing and editing. **Aayush Chaulagain**: supervision, manuscript writing and editing.

## Conflicts of Interest

The authors declare no conflicts of interest.

## Funding

The authors have nothing to report.

## Supporting information




**Supplementary Material**: brb371049‐sup‐0001‐SuppMatt.docx

## Data Availability

The dataset supporting the conclusions of this study is included in the article.
