# Supplementary material for: Efficacy and Safety of Nerinetide in Acute Ischemic Stroke Patients: A Systematic Review, Meta‐Analysis and Meta Regression
Source: Brain Behav. 2025 Nov 10;15(11):e71049. doi: 10.1002/brb3.71049 (PMC12602456; doi:10.1002/brb3.71049)

**Supplementary Table 1.** Search Strategies for different Databases

| **Database** | **Search String** |
| --- | --- |
| PubMed/MEDLINE  (51 Results) | ("nerinetide"[Supplementary Concept] OR "nerinetide"[All Fields]) AND ("acute ischemic stroke"[All Fields] OR "ischemic stroke"[All Fields] OR "acute stroke"[All Fields] OR "stroke"[All Fields]) |
| Cochrane Library  (48 Results) | Nerinetide AND acute ischemic stroke |
| Science Direct  (72 Results) | Nerinetide AND acute ischemic stroke |
| Clinical Trial Govt  (3 results) | Intervention: Nerinetide; Condition: acute ischemic stroke |

**Supplementary Table 2.** Risk of Bias Assessment

|  | Cochrane Risk-of-Bias Tool | | |
| --- | --- | --- | --- |
|  | Bias | Risk of bias | Author judgement |
| Michael D et al 2020 | Random sequence generation (selection bias) | Low Risk | Patients were randomly assigned in a 1:1 ratio using a real-time, internet-based, stratified minimisation procedure, ensuring random allocation. |
|  | Allocation concealment (selection bias) | Low Risk | Allocation was fully masked. The computer-based minimisation system concealed the allocation sequence from trial personnel and participants. |
|  | Blinding of participants and personnel (performance bias) | Low Risk | The study was double-blind; both participants and personnel, including treating clinicians, were blinded to treatment allocation. |
|  | Blinding of outcome assessment (detection bias) | Low Risk | Outcome assessment, including mRS scoring, was conducted by trained, blinded assessors. Imaging was interpreted centrally by a blinded core laboratory. |
|  | Incomplete outcome data (attrition bias) | Low Risk | Only 0.8% of patients had missing outcome data. Imputation of missing data as worst-case values (i.e., poor outcome or death) minimized bias. |
|  | Selective reporting (reporting bias) | Low Risk | All prespecified primary and secondary outcomes were reported. The trial was registered (NCT02930018) and adhered to the protocol. |
|  | Other bias | Low Risk | No other significant sources of bias were identified. The study was well-conducted with robust methods and regulatory oversight. |
| Jim Christenson et al 2025 | Random sequence generation (selection bias) | Low Risk | Participants were randomly assigned in a 1:1 ratio using a computer-generated randomization schedule stratified by center. |
|  | Allocation concealment (selection bias) | Low Risk | Randomization was performed using a secure web-based system managed independently, concealing allocation. |
|  | Blinding of participants and personnel (performance bias) | Low Risk | The study was double-blind; both participants and personnel, including treating clinicians, were blinded to treatment allocation. |
|  | Blinding of outcome assessment (detection bias) | Low Risk | Primary outcomes were assessed by blinded, independent assessors and adjudicated centrally. |
|  | Incomplete outcome data (attrition bias) | Unclear Risk | There was minimal loss to follow-up; outcome data were available for nearly all participants. |
|  | Selective reporting (reporting bias) | Low Risk | All pre-specified outcomes reported in the protocol and registered trial (ACTRN12618001322235) were included in the publication. |
|  | Other bias | Low Risk | No other notable biases identified. The study followed good clinical practice guidelines and was overseen by independent monitors. |
| Michael D Hill et al 2025 | Random sequence generation (selection bias) | Low Risk | Patients were randomly assigned in a 1:1 ratio using a computer-generated randomization sequence, ensuring adequate random allocation. |
|  | Allocation concealment (selection bias) | Low Risk | Randomization was managed by an independent statistician and concealed using a centralized, web-based system. |
|  | Blinding of participants and personnel (performance bias) | Low Risk | The study was double-blind; both participants and personnel, including treating clinicians, were blinded to treatment allocation. |
|  | Blinding of outcome assessment (detection bias) | Low Risk | Outcome assessors were blinded to treatment allocation during follow-up, reducing detection bias. |
|  | Incomplete outcome data (attrition bias) | Low Risk | Follow-up at 90 days was completed for all patients; there were no significant losses to follow-up. |
|  | Selective reporting (reporting bias) | Low Risk | All primary and secondary outcomes described in the protocol and trial registration (NCT02216670) were reported. |
|  | Other bias | Low Risk | No other significant sources of bias identified. Trial was publicly funded and conducted independently. |

**Supplementary Figure 1A.** Regression plot for Mean age

**Supplementary Figure 1B.** Regression plot for Hyperlipidaemia

**Supplementary Figure 1C.** Regression plot for Hypertension

**Supplementary Figure 1D.** Regression plot for Atrial Fibrillation

**Supplementary Figure 1E.** Regression plot for Any past stroke

**Supplementary Figure 1F.** Regression plot for Diabetes

**Supplementary Figure 1G.** Regression plot for Peripheral Vascular Disease

**Figure 2A.** Forest plot for Infarct Volume


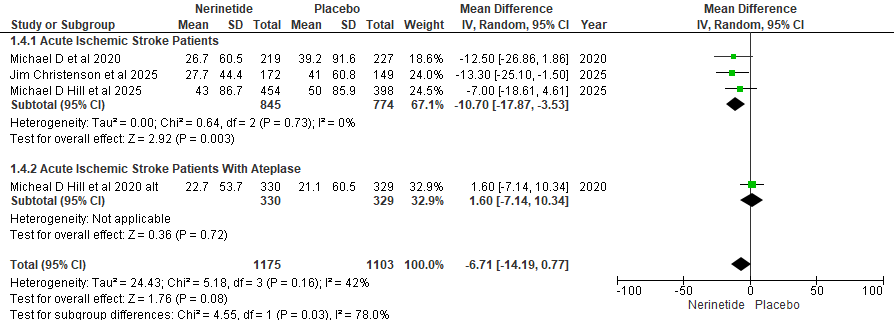


**Figure 2B.** Forest plot for Barthel Index ≥95


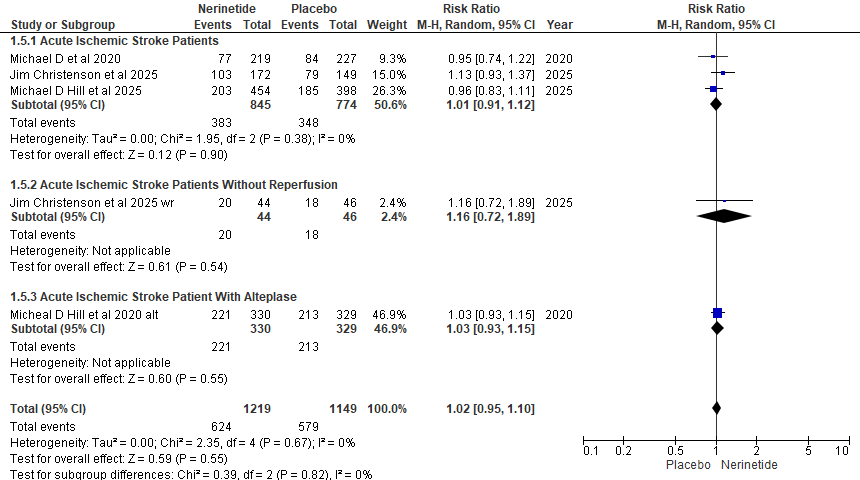


**Figure 2C.** Forest plot for Worsening of stroke


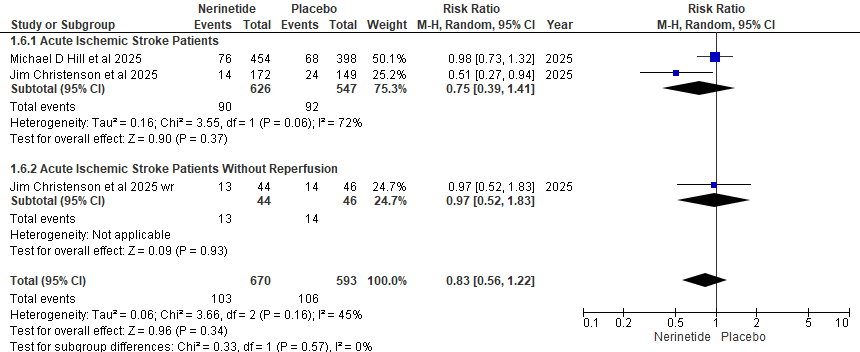


**Figure 2D.** Forest plot for Stroke in evolution


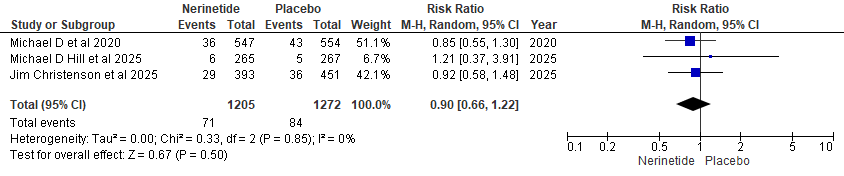


**Figure 2E.** Forest plot for Recurrent or new Ischemic stroke


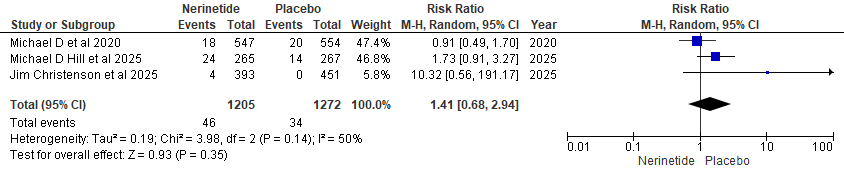


**Figure 2F.** Forest plot for Hypotension


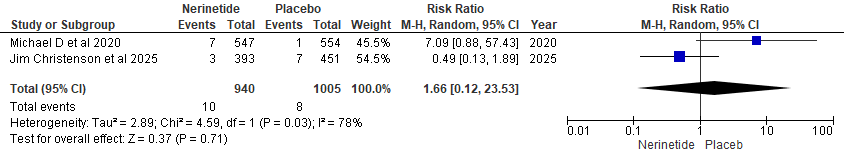


**Figure 2G**. Forest plot for Any Serious Adverse Events


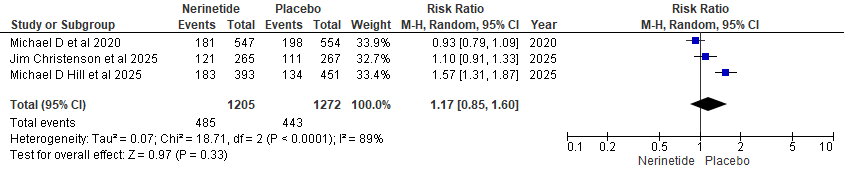


**Figure 2H.** Forest plot for Seizure


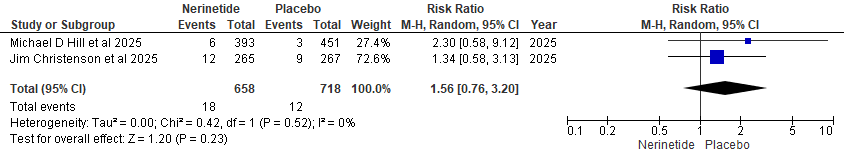


**Figure 2I.** Forest plot for Pulmonary embolism or deep vein thrombosis


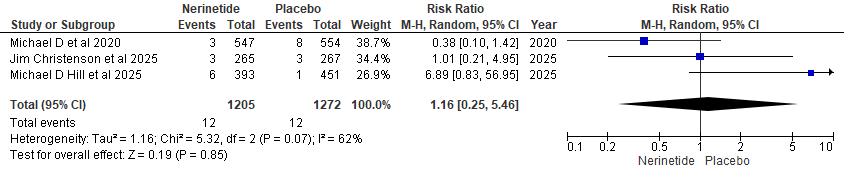

Supplement: Supplementary file 1 — Supplementary Material: brb371049‐sup‐0001‐SuppMatt.docx [file BRB3-15-e71049-s001.docx]
